# Thermoresponsive Cationic Polymers: PFAS Binding Performance under Variable pH, Temperature and Comonomer Composition

**DOI:** 10.3390/gels8100668

**Published:** 2022-10-18

**Authors:** E. Molly Frazar, Anicah Smith, Thomas Dziubla, J. Zach Hilt

**Affiliations:** Department of Chemical and Materials Engineering, University of Kentucky, Lexington, KY 40506, USA

**Keywords:** thermoresponsive, PFAS, water remediation, cationic hydrogel

## Abstract

The versatility and unique qualities of thermoresponsive polymeric systems have led to the application of these materials in a multitude of fields. One such field that can significantly benefit from the use of innovative, smart materials is environmental remediation. Of particular significance, multifunctional poly(N-isopropylacrylamide) (PNIPAAm) systems based on PNIPAAm copolymerized with various cationic comonomers have the opportunity to target and attract negatively charged pollutants such as perfluorooctanoic acid (PFOA). The thermoresponsive cationic PNIPAAm systems developed in this work were functionalized with cationic monomers *N*-[3-(dimethylamino)propyl]acrylamide (DMAPA) and (3-acrylamidopropyl)trimethylammonium chloride (DMAPAQ). The polymers were examined for swelling capacity behavior and PFOA binding potential when exposed to aqueous environments with varying pH and temperature. Comonomer loading percentages had the most significant effect on polymer swelling behavior and temperature responsiveness as compared to aqueous pH. PFOA removal efficiency was greatly improved with the addition of DMAPA and DMAPAQ monomers. Aqueous pH and buffer selection were important factors when examining binding potential of the polymers, as buffered aqueous environments altered polymer PFOA removal quite drastically. The role of temperature on binding potential was not as expected and had no discernible effect on the ability of DMAPAQ polymers to remove PFOA. Overall, the cationic systems show interesting swelling behavior and significant PFOA removal results that can be explored further for potential environmental remediation applications.

## 1. Introduction

Stimuli-responsive polymers have long been an attractive option for a wide range of applications due to the transformations exhibited upon exposure to external stimuli [1]. Specifically, temperature-responsive polymers have been reported as especially useful in the fields of biomedicine [2,3], drug delivery [3,4,5], microfluidics [3,6], environmental remediation [7,8] and separations [9]. The unique qualities of these types of systems are many-fold but the most remarkable is a reversible phase change that occurs at a critical solution temperature in aqueous solvent. This behavior can be attributed to a disruption of intra- and intermolecular interactions that cause the polymer to expand or collapse [10]. Thermoresponsive hydrogels have the ability to swell in aqueous environments without dissolving, due to a volume phase transition around their characteristic critical temperature.

**Figure 1:**
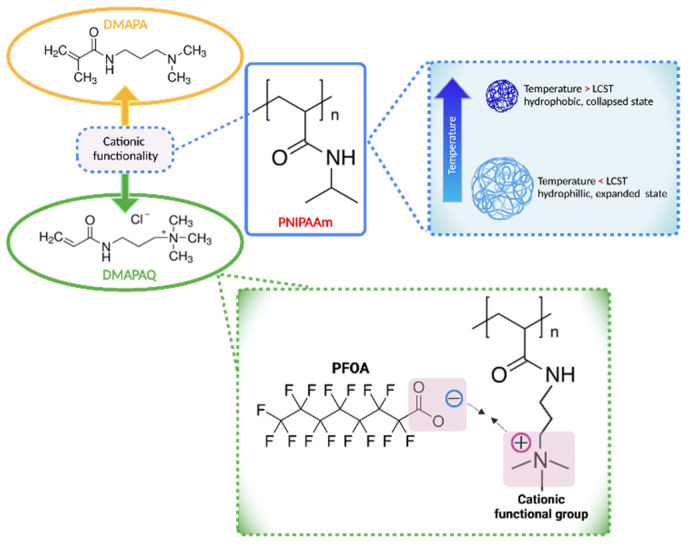
PNIPAAm-based polymers undergo phase changes from hydrophilic to hydrophobic at a lower critical transition temperature (LCST~32 °C) and can be modified with various comonomers to yield functionalities that attract environmental pollutants such as PFOA.

The most extensively studied thermoresponsive hydrogels are those based on poly(N-isopropylacrylamide) (PNIPAAm). PNIPAAm polymers undergo phase transitions from hydrophilic to hydrophobic at a lower critical transition temperature (LCST) around 32 °C [11,12]. As illustrated in Figure 1, PNIPAAm polymer chains are hydrated and begin to expand when external temperatures drop below the LCST, resulting in a swollen polymer state. Conversely, PNIPAAm polymer chains become hydrophobic if external temperatures rise above the LCST, causing the polymer network to collapse [12]. This phenomenon has been attributed to hydrogen bond formation/destruction between water molecules and the amide groups present in PNIPAAm [12,13]. Research reporting on the application of such stimuli-responsive hydrogels for environmental remediation purposes is not novel and several well-organized reviews discuss synthesis and application specifics [14,15,16]. In short, because thermoresponsive hydrogels, such as PNIPAAm, exhibit hydrophilic behavior at a certain temperature range, many aqueous contaminants are allowed to easily diffuse into the hydrogel-based sorbent. This type of binding model provides an alternative to that demonstrated by activated carbon or other traditional sorbents [16]. In addition, thermoresponsive stimulus changes can aid in the removal of environmental contaminants through expansion to drive sorption and contraction to drive desorption [17]. Copolymerization with various comonomers can yield polymers containing functionalities that modify the network properties. The addition of comonomers to PNIPAAm systems can cause shifts in the polymer LCST, subsequently affecting swelling behavior [18]. These materials, however, can be designed to exhibit characteristic properties (e.g., electrostatic interactions with contaminants of interest, such as ubiquitous per- and polyfluoroalkyl substances, PFAS), and the swelling/shrinking properties of these systems have created a platform for the development of useful sorbents that can potentially target and remove contaminants when needed [19].

One category of environmental contaminants that could be targeted through application of stimuli-responsive hydrogels is poly- and perfluoroalkyl substances (PFAS). PFAS are a class of an ever-growing number of compounds (now believed to encompass approximately 12,000 compounds) and have been frequently used for their stain and water resistant properties [20]. They have been dubbed as “forever chemicals” due to their extremely persistent nature. In fact, no environmental half-life has been established thus far. Numerous research studies have linked human PFAS exposure to shocking health consequences such as several types of cancer, thyroid, kidney and liver disease, cholesterol dysregulation, developmental and reproductive issues, and immune suppression [21,22,23,24,25,26,27,28]. As such, there has never been a more suitable time for innovative and renewable materials research to address the current state of environmental pollution. Facile functionalization of thermoresponsive hydrogels can produce materials with extremely desirable physical characteristics for exactly this type of application [19].

The thermoresponsive cationic polymers examined in this work have been synthesized by free radical polymerization of a temperature-responsive platform of PNIPAAm crosslinked with *N-N*’-methylenebis(acrylamide) (NMBA) and various cationic comonomers: (1) dimethylamino propyl acrylamide (DMAPA), and its quaternized sister form, (2) dimethylamino propyl acrylamide, methyl chloride quaternary (DMAPAQ). Herein, we explore the effect of polymer composition, environmental pH, and temperature on hydrogel swelling behavior and fluorinated contaminant binding affinity. Perfluorooctanoic acid (PFOA) was chosen as a model PFAS contaminant and binding studies were conducted by treating PFOA-spiked water samples with the synthesized polymers. Cationic monomers are used to functionalize the polymers to equip them with positively charged moieties that interact and bond with deprotonated PFOA moieties through electrostatic interactions. It was hypothesized that increased addition of cationic comonomer content hinders thermoresponsive behavior and swelling capacity of the PNIPAAm hydrogels while conversely enhancing polymer PFOA affinity. Binding studies conducted at temperatures below polymer LCST are expected to result in higher removal efficiencies than those conducted above LCST temperature. 

## 2. Results and Discussion

Various crosslinked NIPAAm-based copolymers with varying cationic comonomer type and concentrations were successfully synthesized by free-radical polymerization along with a crosslinked PNIPAAm hydrogel that did not contain a cationic comonomer. Thermoresponsive cationic polymers were synthesized by copolymerization of NIPAAm with DMAPA or DMAPAQ, and successful incorporation of the cationic comonomers was confirmed by FTIR analysis (Figures S1 and S2).

### 2.1. Swelling Behavior

#### 2.1.1. Effect of Aqueous pH 

**Figure 2:**
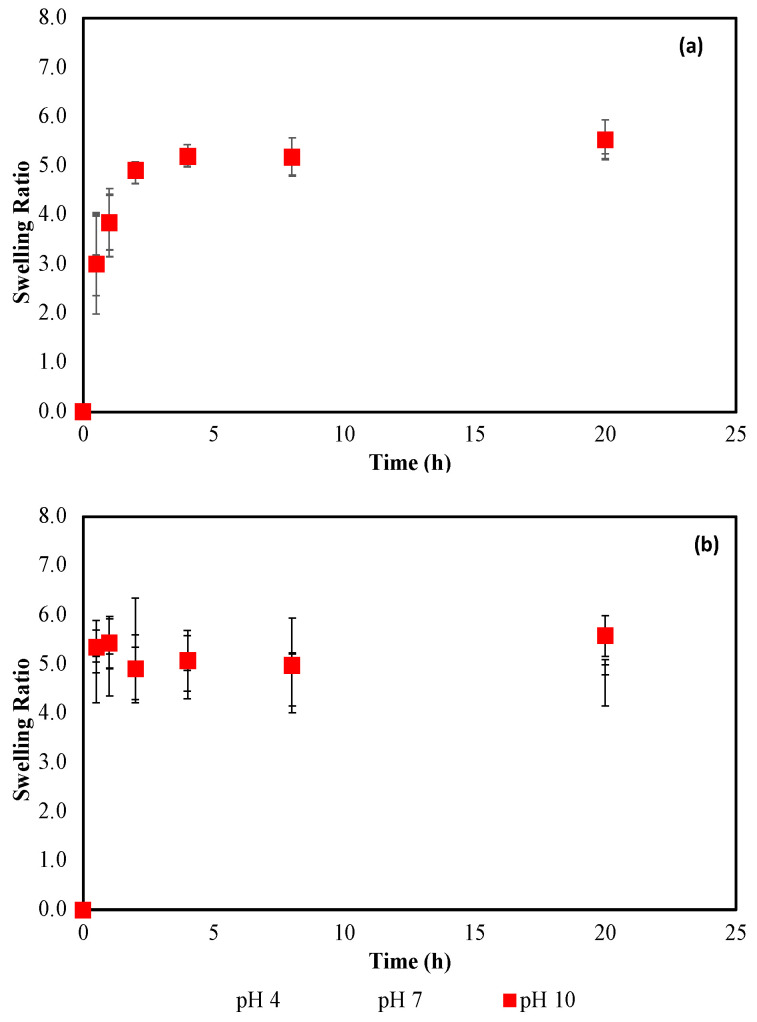
Kinetic swelling behavior of crosslinked PNIPAAm (95 mol%) in various aqueous pH: (**a**) buffered DI-H_2_O at 20 °C and I = 0.15 M, (**b**) titrated DI-H_2_O at 20 °C; n = 3, error bars represent ± STD.

As illustrated in Figure 2, aqueous pH had little discernible effect on PNIPAAm equilibrium mass swelling ratio when examined in both buffered and titrated solutions at 20 °C (held in isothermal water bath). Additionally, the difference in swelling ratio for PNIPAAm in both aqueous systems is not statistically significant and generally average out to be *Q_eq,buff_* = 5.4 ± 0.3 over the three pH systems (Table 1). Similarly, titrated aqueous solutions had little effect on the swelling ratio of PNIPAAm at various pH (average *Q_eq,tit_* = 5.0 ± 0.3), with the only slight difference occurring between the pH = 4 and 10 systems (Figure 2b). This could be attributed to the effect of increased hydrogen ion interaction with the isopropyl and amino functional groups present in PNIPAAm, allowing for more hydrated polymer chains. It can be noted, however, when comparing swelling kinetics between gels placed in a buffered vs. titrated solution, equilibrium is achieved much more rapidly for the titrated solution gels (Figure 2) likely due to increased electrolyte presence in the buffered solutions that can interact with the polymer chains and slow the hydration process. 

**Figure 3:**
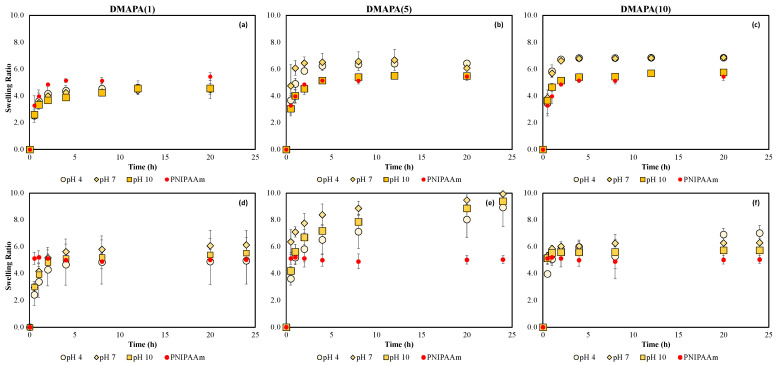
Kinetic swelling behavior of DMAPA hydrogels in the presence of varying pH aqueous solutions at 20 °C: buffered swelling behavior of (**a**) DMAPA(1), (**b**) DMAPA(5), (**c**) DMAPA(10), and titrated DI-H_2_O swelling behavior of (**d**) DMAPA(1), (**e**) DMAPA(5), (**f**) DMAPA(10). Red circles indicate PNIPAAm swelling averages; n = 3, error bars represent ± STD.

**Figure 4:**
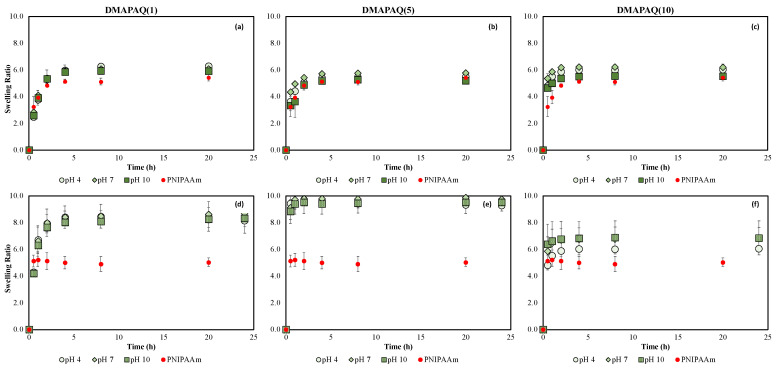
Kinetic swelling behavior of DMAPAQ hydrogels in the presence of varying pH aqueous solutions at 20 °C: buffered swelling behavior of (**a**) DMAPAQ(1), (**b**) DMAPAQ(5), (**c**) DMAPAQ(10), and titrated DI-H_2_O swelling behavior of (**d**) DMAPAQ(1), (**e**) DMAPAQ(5), (**f**) DMAPAQ(10). Red circles indicate PNIPAAm swelling averages; n = 3, error bars represent ± STD.

At the low comonomer loading percentage, pH has little effect on DMAPA(1) and DMAPAQ(1) hydrogels (Figure 3a,d and Figure 4a,d) at 20 °C, just as is seen with the PNIPAAm gels. For DMAPA(5) and DMAPA(10) loading, however, swelling ratio decreases with increasing pH. This is likely due to the amide functional group present in DMAPA gels, which would potentially become deprotonated at high pH values and thus tend to result in more significant dehydration of the polymer chains. Additionally, note the behavior of DMAPA(5) and DMAPA(10) in the temperature-dependent swelling study, where pH 10 resulted in hydrogel behavior similar to that of PNIPAAm, even as swelling ratios for the pH 4 and pH 7 systems reached much higher values, indicating limited collapse and a significant loss in thermoresponsive behavior (Figures S3 and S4). This trend, however, is not as significantly noticed for the DMAPAQ polymers, although DMAPAQ(5) gels do show similar inclination (Figure S5).

DMAPAQ hydrogels show similar results to those of the DMAPA polymers, although they do not exhibit quite the drastic pH dependence (Figure 4. Swelling ratios of higher loading gels decrease when pH is raised to 10 for all temperatures (Figure S5). Unlike the DMAPA gels, however, thermoresponsive behavior only seems to be maintained for DMAPAQ(5) at pH 10 and not for DMAPAQ(10) at pH 10 (Figures S4 and S5). This would suggest that the quaternary ammonium functional group present in DMAPAQ allows the polymer chain to stay hydrated even as hydrogen ion concentration decreases, bringing insight into how important amino functional groups are for polymer hydration/dehydration. 

Comparisons between swelling studies conducted in buffered versus titrated solutions are reported to be significant, as shown in Figure 2, Figure 3 and Figure 4. Swelling dependence on solution pH was insignificant for PNIPAAm, DMAPA, and DMAPAQ gels in titrated solutions, and only showed some discernible difference in buffered solution for DMAPA(5) and DMAPA(10) gels. 

#### 2.1.2. Effect of Comonomer Composition 

To examine the impact of cationic comonomer addition, crosslinked PNIPAAm (95 mol%) was used as a control for all swelling studies, and as an effective visual comparison, the averaged PNIPAAm swelling ratios are included on all other copolymer swelling graphs as red circle markers. DMAPA(1) exhibits diminished swelling behavior in buffered solution, *Q_eq,buff,pH7_* = 4.5 ± 0.7 (Figure 3a), as opposed to PNIPAAm *Q_eq,buff,avg_* = 5.4 ± 0.3, whereas DMAPAQ(1) is seen to have slightly increased swelling behavior at *Q_eq,buff,pH7_* = 6.1 ± 0.2 (Figure 4a). Interestingly, out of all the examined systems, the addition of DMAPA(5) and DMAPAQ(5) appeared to have the greatest swelling when placed in the nonbuffered solution where polymer swelling capacity was significantly increased. It is unexpected that the 10 mol% loading would not further increase the swelling of these systems, since comonomer addition is expected to increase polymer hydrophilicity. It is our speculation that interactions between the comonomer and other functionalities present in the PNIPAAm and/or NMBA chains are hindering polymer swelling capacity. Using pH swings in congruence with temperature shifts could offer some additional functionality to the DMAPA and DMAPAQ monomers. It was observed, however, that higher aqueous pH values resulted in thermoresponsive swelling similar to that of PNIPAAm. For instance, for DMAPA(10) hydrogels in a buffered aqueous environment of pH = 7, a swelling ratio of *Q_eq,buff,pH7_* = 6.9 ± 0.2 was observed, but the gel was only able to contract back down to *Q_eq,buff,pH7_* = 4.2 ± 0.4 at 60 °C if left in the same pH environment. However, if the aqueous environment was altered to pH = 10 after the gel had reached equilibrium, it collapsed to *Q_eq,buff,pH10_* = 1.9 ± 0.3 at 60 °C (Figure 3c and Figure S4).

Comparison of the DMAPAQ gels placed in buffered versus titrated solutions reveals an interesting variance. The swelling ratios for DMAPAQ(1) and DMAPAQ(5) polymers were significantly stifled in buffered solutions and remained similar to that of pure PNIPAAm, likely due to interactions with electrolytes present in the buffer solution that were not present in the titrated solutions (Figure 4a,b,d,e). DMAPAQ(1)-titrated swelling ratios are about 20% higher than those of the buffered solution, while DMAPAQ(5)-titrated swelling ratios are around 60% higher than their buffered counterparts. Interestingly, this trend is not as prominent for the higher-loading DMAPAQ(10) gels (Figure 4c,f).

Ultimately, the results reported here show a greater swelling deviation from PNIPAAm for DMAPA and DMAPAQ when examined in titrated solution rather than buffered solution, particularly for DMAPAQ gels.

### 2.2. PFOA Binding Affinity

**Figure 5:**
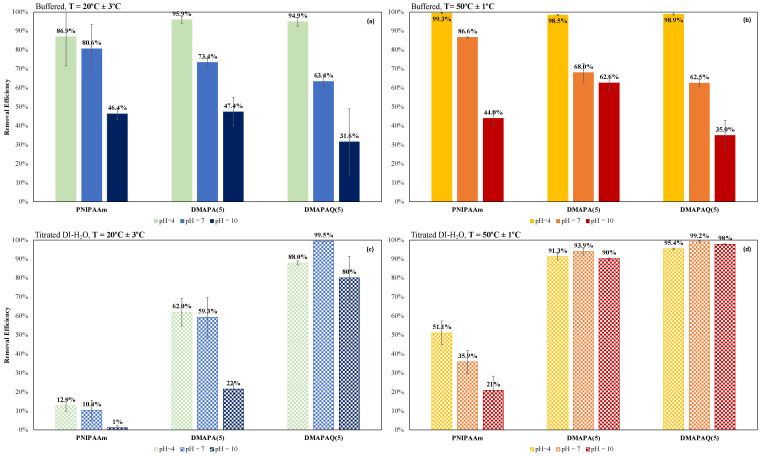
PFOA removal efficiency of PNIPAAm, DMAPA(5), and DMAPAQ(5) hydrogels at various aqueous pH values after 20 h: (**a**) buffered aqueous solution at T = 20 °C, (**b**) buffered aqueous solution at T = 50 °C, (**c**) titrated aqueous solution at T = 20 °C, and (**d**) titrated aqueous solution at T = 50 °C; n = 3, error bars represent ± STD.

To investigate the role of amine-functionalized monomers in thermoresponsive polymer sorbents on legacy PFAS uptake, PFOA removal was evaluated in batch experiments using a higher concentration ([PFOA]_0_ = 200 μg L^−1^) than those found in most contaminated water sources, along with a relatively high polymer concentration (2500 mg L^−1^) (Figure 5). Hydrogel affinity toward PFOA was demonstrated to be significantly affected by pH when placed in buffered solutions (Figure 5a,b). All polymeric systems in buffered solutions showed high removal efficiencies (>90%) for PFOA at pH = 4. This is most likely due to the nature of PFOA itself, which usually assumes a deprotonated state. Increasing pH presumably results in decreased electrostatic interactions between the negatively charged contaminant and positively charged polymer. Furthermore, although it was expected that higher adsorption would occur at temperatures below the PNIPAAm LCST, there appears to be no significant difference between binding studies conducted at 20 °C versus those conducted at 50 °C in buffered solution. DMAPA(5) was the only system that hinted at a thermoresponsive binding trend by achieving removal efficiencies of 73.4% at pH 7 when examined at 20 °C, which is below polymer LCST. Removal efficiency was decreased to 68.0% at pH 7 when binding temperature was above LCST. The addition of the cationic comonomers to PNIPAAm gels did not have a significant impact on PFOA binding affinity when the gels were examined in buffered solution, despite evidence that binding mostly occurs through ionic interactions between anionic PFOA heads and cationic polymer moieties. In fact, PNIPAAm outperformed DMAPAQ systems at buffered aqueous pH 7 and 10. DMAPA gels had slightly higher removal efficiencies than the other systems at pH 10, and this was one of the only instances where the 50 °C binding study showed higher removal than the 20 °C binding study. A higher percentage of removal efficiency achieved by PNIPAAm could most likely be attributed to ionic attraction of the hydrophilic carboxylic head of PFOA to the hydrophilic secondary amine functionality in PNIPAAm.

Removal efficiencies for the DMAPA(5) and DMAPAQ(5) systems significantly improved when examined in titrated aqueous conditions as compared to those reported in buffered solutions. For example, DMAPA(5) examined in aqueous pH 7 at 50 °C had PFOA removal of 93.9% in titrated solution as compared to 68.0% in buffered solution. Similarly, DMAPAQ(5) saw an increase from 62.5% PFOA removal to 99.2% removal in the same conditions (Figure 5). As expected, the quaternary amine containing DMAPAQ performs consistently well across all pH values. PNIPAAm, DMAPA, and DMAPAQ gels achieved lower removal efficiencies when binding studies were conducted at 20 °C, contradictory to what was originally hypothesized. For gels in titrated aqueous solutions of pH 7 at both 20 and 50 °C, DMAPAQ(5) achieved near 100% removal (within error), which would bring PFOA measurements within acceptable range of EPA lifetime health advisory limits [29]. Absolute capture is an important measure when considering environmental contaminants such as PFOA. Determining an equilibrium state and binding equilibrium constant for the hydrogel sorbents are crucial factors for reporting reliable binding measurements. Additional studies with these materials are needed to establish appropriate contact time and concentration ranges.

## 3. Conclusions

In this work, a variety of thermoresponsive cationic hydrogels were successfully synthesized via free radical polymerization. The effect of pH on hydrogel swelling behavior was found to be insignificant for PNIPAAm and hydrogels containing loading percentages of 1 and 5 mol% cationic comonomer. Inclusion of cationic comonomers, however, did alter hydrogel swelling capacity, mostly due to losses in thermoresponsive behavior as the comonomer amount was increased. For all three cationic comonomer systems, high loading percentages led to significantly higher swelling ratios that also corresponded to a decrease in the inability to collapse as temperature was increased above the LCST. The only exceptions to this observed behavior were seen at aqueous pH = 10 where DMAPA(10) and DMAPAQ(5) behaved similarly to that of PNIPAAm (Figures S4 and S5). Sorption of PFOA was inversely related to buffered aqueous pH, while cationic monomer type had little noticeable consequence in the buffered solutions. A stark contrast is observed, however, when binding studies are conducted in titrated aqueous environments, indicating that buffer selection can deeply hinder contaminant removal efficiency because of competitive electrostatic interactions. These insights gained from hydrogel performance under variable pH, buffer, temperature, and comonomer composition provide us with a deeper understanding of which polymer functionalities are most beneficial when designing materials for PFAS remediation in aqueous environments.

## 4. Materials and Methods

### 4.1. Materials

*N*-isopropylacrylamide (NIPAAm, Sigma-Aldrich, St. Louis, MO, USA, 97%), *N*-[3-(dimethylamino)propyl]acrylamide (DMAPA, TCI, ≥98.0%), (3-acrylamidopropyl)trimethylammonium chloride (DMAPAQ, Sigma-Aldrich, St. Louis, MO, USA, 75 wt% in H_2_O), crosslinker *N-N*’-methylenebis(acrylamide) (NMBA, BeanTown Chemical, 99%), initiator ammonium persulfate (APS, Sigma-Aldrich, St. Louis, MO, USA, ≥98%), catalyst *N,N,N’,N’*-tetramethylethylenediamine (TEMED, VWR, ≥98%), perfluorooctanoic acid (PFOA, Sigma-Aldrich, St. Louis, MO, USA, 95%) were used as received. Ultrapure water (resistivity 18.2 MΩ) was used in all synthesis reactions and subsequent experiments.

### 4.2. Hydrogel Synthesis

Preparation of PNIPAAm hydrogel systems was conducted via free radical polymerization reactions (Table 1). The calculated amounts of NIPAAm and cationic comonomer were dissolved in ultrapure water (2 mL) with feed ratios of 95/0, 94/1, 90/5, or 85/10 mol% and crosslinker NMBA kept constant at 5 mol%. A 0.5 mg/mL initiator stock solution was prepared by dissolving 50 mg of APS in 1 mL ultrapure water and was added to the reactant solution at 0.1 wt% combined weight of NIPAAm, comonomer, and NMBA. The catalyst TEMED was added at 2 wt% combined weight of NIPAAm, comonomer, and NMBA. Vortex mixing was conducted for 10 s before the reactant solution was transferred to glass templates to create hydrogel sheets with dimensions of 1 mm by 12 mm by 24 mm. The polymerization reaction was allowed to proceed for 24 h at ambient conditions. The synthesized hydrogels were immersed in ultrapure water for an additional 24 h at ambient temperature to ensure removal of any unreacted monomers, initiator, or catalyst, during which the wash water was replaced with fresh water at least 3 times. After washing, the polymers were cut into small rectangular pieces and oven dried at 75 °C for 24 h. Dried polymers were either stored as is or ground with mortar and pestle into fine powder to be used for subsequent swelling and binding studies. 

### 4.3. Hydrogel Characterization

#### 4.3.1. FTIR Analysis 

Attenuated total reflectance Fourier transform infrared (ATR-FTIR) was used to confirm successful incorporation of the cationic comonomers into the synthesized hydrogels with a Varian Inc. 7000e spectrometer. Dried samples were placed on a diamond ATR crystal and spectrums were obtained between 700 and 4000 cm^−1^.

#### 4.3.2. Kinetic Swelling Study 

Swelling kinetics of each hydrogel were determined via gravimetric analysis in both buffered and titrated DI-H_2_O aqueous solutions. Buffered solutions were prepared at pH = 4 and 7 using a phosphate citrate buffer and at pH = 10 using an ammonia buffer. Titrated DI-H_2_O solutions were prepared through slow addition of either 1 M NaOH or 2 M HCl to DI-H_2_O to achieve aqueous pH of 4, 7, or 10. An approximately 10.0 mg piece of dry gel was immersed in 5 mL of aqueous solution at 20 °C in an isothermal water bath and measurements were taken at regular intervals of 0.5, 1, 2, 4, 8, 12 and 20 h. To do so, each sample was removed from the solution, gently patted with a Kimwipe to remove excess surface water, and quickly weighed on an analytical balance. The equilibrium mass swelling ratio (*Q_eq_*) of the hydrogel in buffered aqueous solution was calculated according to Equation (1):

(1) $$Q_{eq}=\frac{m_{s}}{m_{d}}$$

where m_s_ (mg) and m_d_ (mg) are the sample weight of the swollen and dried hydrogel samples, respectively. 

#### 4.3.3. Temperature-Dependent Swelling Study

Temperature responsiveness of each hydrogel was examined by allowing an approximately 10.0 mg piece of dry gel to equilibrate in 5 mL of aqueous solution (pH = 4, 7, or 10 of buffered solution as described above) for 24 h at various solution temperatures. Swelling ratios were measured at temperatures of 10, 20, 25, 30, 35, 40, and 50 °C. Mass measurements were collected by the same method described in the kinetic swelling study section above. The mass swelling ratio was calculated using Equation (1). 

#### 4.3.4. PFOA Binding Affinity

Environmental remediation proof-of-concept experiments were conducted by examining the PFOA binding potential of the synthesized polymers through straightforward equilibrium binding studies. Approximately 2.5 mg/mL of dried granulated sorbent (only cationic polymers DMAPA(5) and DMAPAQ(5) were examined along with PNIPAAm and no sorbent as a negative control) was added to aqueous solutions (pH = 4, 7, or 10 of buffered or titrated solutions as described above) spiked with 200 ppb PFOA in glass vials. The system was then agitated on an orbital shaker for 20 h at either 20 or 50 °C. Subsequently, all samples (including controls) were filtered via 0.2 μm syringe tip filter before analysis via liquid chromatography mass spectrometry (LC–MS/MS). Ultraperformance liquid chromatography (UPLC) coupled with electrospray ionization tandem mass spectrometry was used for analysis of PFOA concentration. Instrumentation included a bench-top binary prominence Shimadzu chromatograph (Model: LC-20 AD) equipped with a SIL 20 AC HT autosampler interfaced with an AB SCIEX Flash Quant mass spectrometer (MS/MS) (Model: 4000 Q TRAP). Limit of detection (LOD) for target analytes were 0.25 ng/L at S/N ¼ 4. Seven calibration points with linear dynamic range (LDR) were within 2.5–320 ng/mL and had R^2^ values of 0.99968. For all PFAS binding experiments, the pollutant removal efficiency by the hydrogel sorbents was calculated as:

(2) $$Removal\;Percentage\;\left(\%\right)=\frac{C_{0}-C_{t}}{C_{0}}\;\times \;100$$

where C_0_ (µg L^−1^) is the initial concentration of PFAS and C_t_ (µg L^−1^) is the concentration of PFAS at time (*t*). The initial concentration C_0_ was obtained from the average concentration of negative control samples to account for loss of pollutant from experimental conditions.

## Figures and Tables

**Table 1:** Comonomer loading percentages for polymer synthesis via free radical polymerization with corresponding equilibrium swelling ratios at 20 °C for various aqueous environments and pH values. Crosslinker loading was consistent for all systems at 5 mol% NMBA; n = 3, numbers in parenthesis represent +/− STD.

Polymer ID	Cationic Comonomer	Comonomer Loading (mol%)	NIPAAm Loading (mol%)	Q_eq,buff_ pH 4	Q_eq,buff_ pH 7	Q_eq,buff_ pH 10	Q_eq,tit_ pH 4	Q_eq,tit_ pH 7	Q_eq,tit_ pH 10
PNIPAAm	--	--	95	5.4(0.2)	5.4(0.2)	5.5(0.4)	4.6(0.4)	4.9(0.1)	5.6(0.4)
DMAPA(1)	DMAPA	1	94	4.5(0.4)	4.5(0.7)	4.6(0.0)	5.0(1.7)	6.1(1.1)	5.5(0.4)
DMAPA(5)		5	90	6.4(0.2)	6.1(0.1)	5.5(0.1)	8.9(1.4)	9.9(0.6)	9.4(0.6)
DMAPA(10)		10	85	6.9(0.1)	6.9(0.2)	5.8(0.0)	7.0(0.6)	6.3(0.3)	5.7(0.6)
DMAPAQ(1)	DMAPAQ	1	94	6.3(0.2)	6.1(0.2)	5.9(0.2)	8.2(1.0)	8.7(1.0)	8.3(0.2)
DMAPAQ(5)		5	90	5.5(0.1)	5.8(0.1)	5.2(0.2)	9.3(0.1)	9.7(0.6)	9.5(0.6)
DMAPAQ(10)		10	85	6.1(0.1)	6.2(0.2)	5.5(0.1)	6.1(0.1)	6.8(0.8)	6.9(1.3)

## Data Availability

The data presented in this study are available on request from the corresponding author.

## Supplementary Materials

The following supporting information can be downloaded at: https://www.mdpi.com/article/10.3390/gels8100668/s1, Figure S1: FTIR spectra for DMAPA hydrogels; Figure S2: FTIR spectra for DMAPAQ hydrogels; Figure S3: Equilibrium temperature-responsive swelling behavior of crosslinked PNIPAAm (95 mol%) in various buffered aqueous pH solutions at *t* = 24 h; n = 3, error bars represent +/− STD; Figure S4: Equilibrium temperature-responsive swelling behavior of crosslinked DMAPA hydrogels in various pH buffered aqueous solutions at *t* = 24 h: (a) DMAPA(1), (b) DMAPA(5), and (c) DMAPA(10). Red circles indicate PNIPAAm swelling averages; N = 3, error bars represent +/− STD; Figure S5: Equilibrium temperature-responsive swelling behavior of crosslinked DMAPAQ hydrogels in various pH buffered aqueous solutions at *t* = 24 h: (a) DMAPAQ(1), (b) DMAPAQ(5), and (c) DMAPAQ(10). Red circles indicate PNIPAAm swelling averages; n = 3, error bars represent +/− STD.
