# Supplementary material for: Thermoresponsive Cationic Polymers: PFAS Binding Performance under Variable pH, Temperature and Comonomer Composition
Source: Gels. 2022 Oct 18;8(10):668. doi: 10.3390/gels8100668 (PMC9602350; doi:10.3390/gels8100668)
Supplement: Supplementary file 1 [file gels-08-00668-s001.zip › gels-1953575-supplementary.pdf]

Supporting Information

Thermoresponsive Cationic Polymers: PFAS Binding Performance under Variable pH, Temperature and Comonomer Composition

FTIR analysis

Attenuated total reflectance Fourier transform infrared (ATR-FTIR) was used to confirm successful incorporation of the cationic comonomers into the synthesized hydrogels with a Varian Inc. 7000e spectrometer. Dried samples were placed on a diamond ATR crystal and spectrums were obtained between 700 and 4,000 cm^{-1}.

Temperature dependent swelling study

Temperature responsiveness of each hydrogel was examined by allowing an approximately 10.0 mg piece of dry gel to equilibrate in 5 mL of aqueous solution (pH = 4, 7 or 10 of buffered solution as described above) for 24 h at various solution temperatures. Swelling ratios were measured at temperatures of 10, 20, 25, 30, 35, 40 and 50 °C. Mass measurements were collected by the same method described in the kinetic swelling study section above. The mass swelling ratio (Q_{eq}) was calculated using:

$$Q_{eq} = \frac{m_s}{m_d}$$

Additional Figures

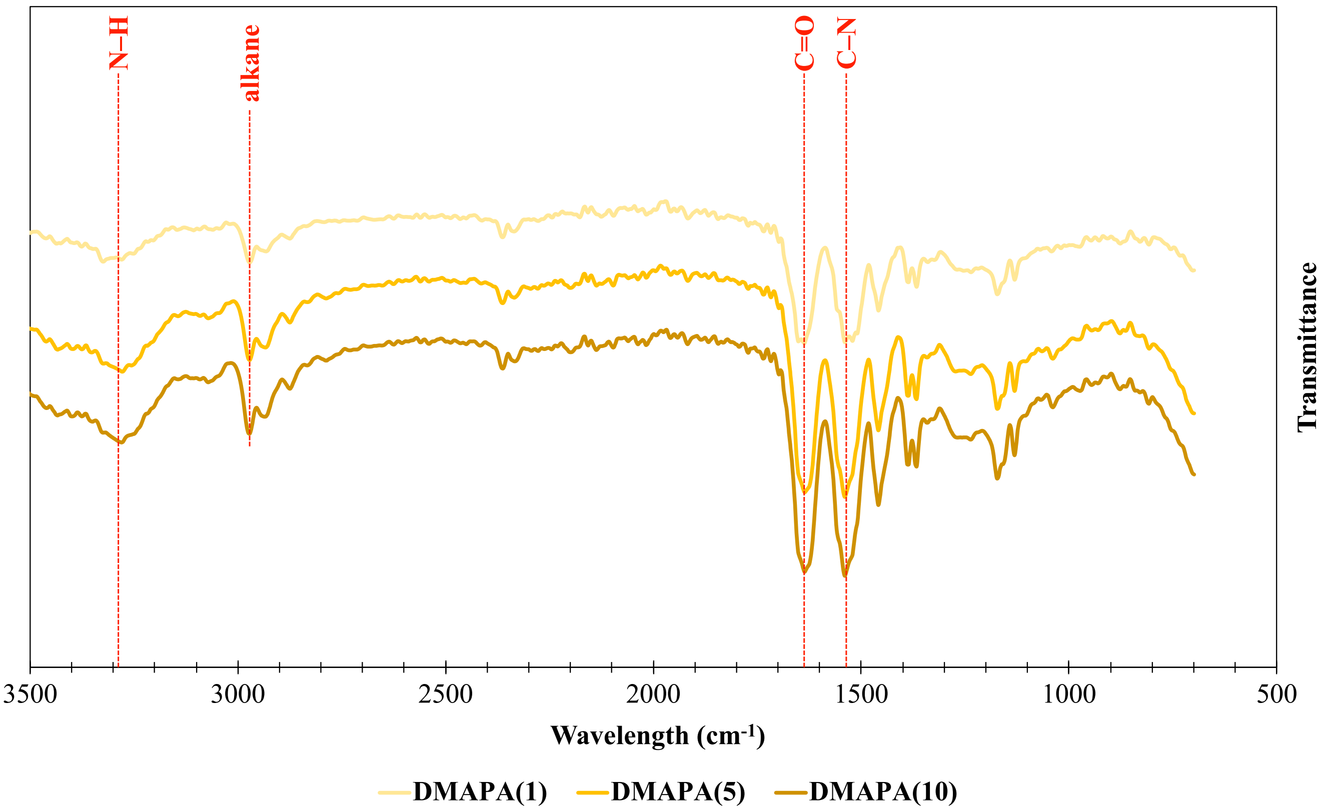

Figure S1. FTIR spectra for DMAPA hydrogels.

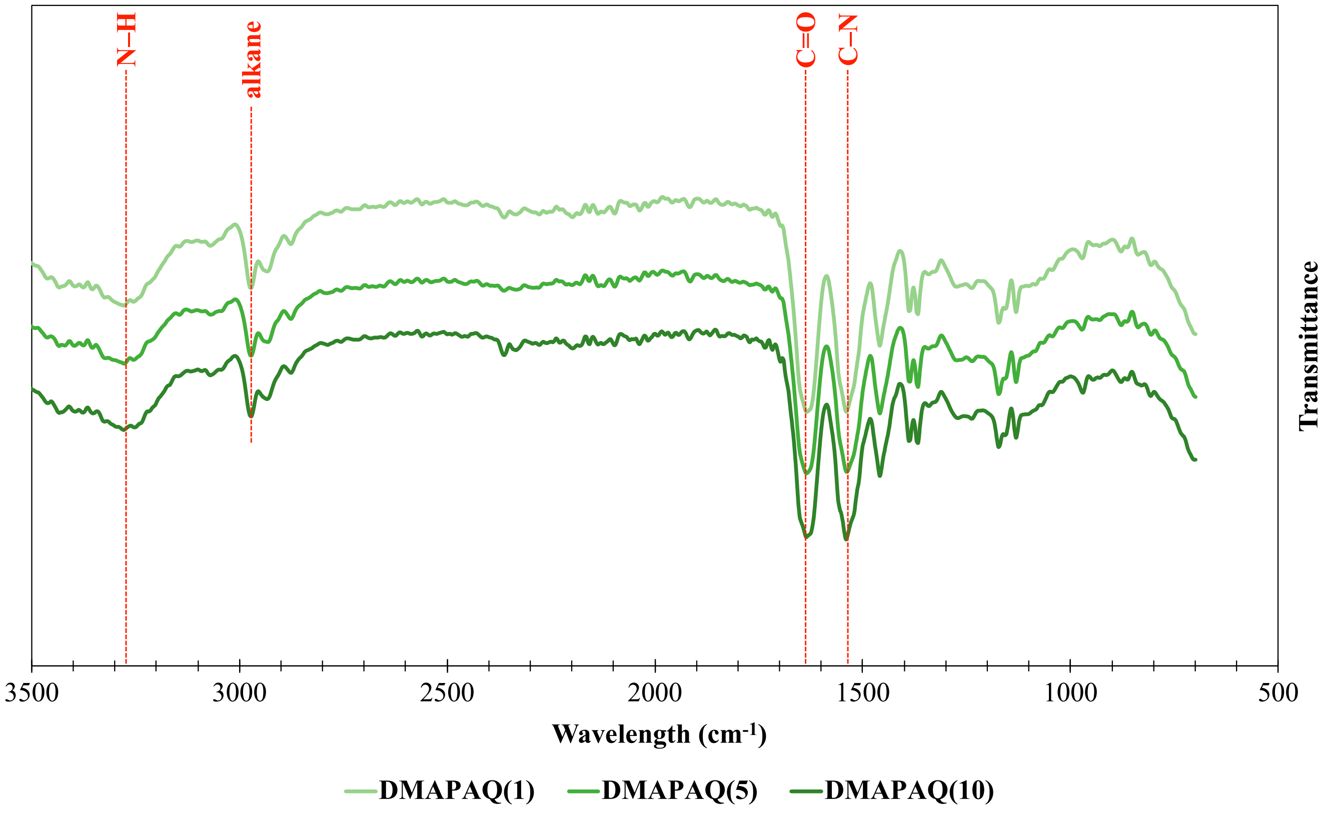

Figure S2. FTIR spectra for DMAPAQ hydrogels.

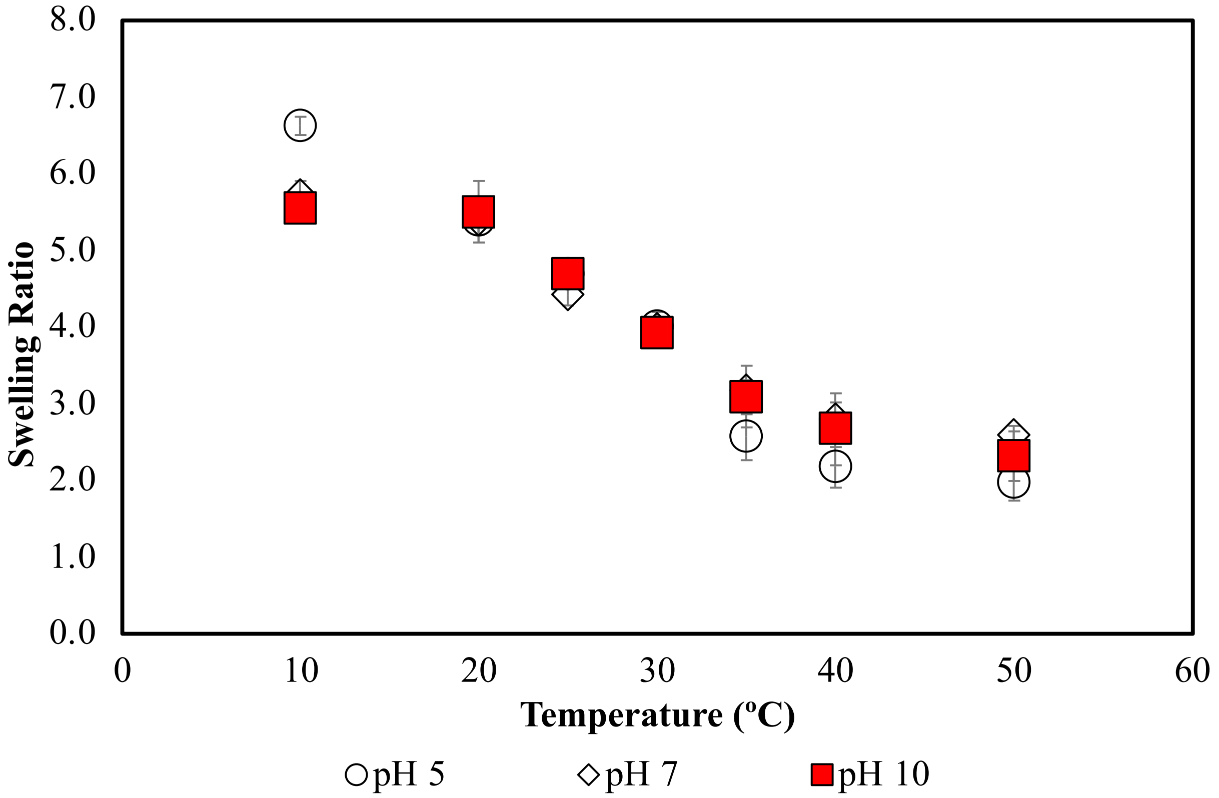

Figure S3. Equilibrium temperature responsive swelling behavior of crosslinked PNIPAAm (95 mol%) in various buffered aqueous pH solutions at t = 24 h. N = 3, error bars represent +/- STD.

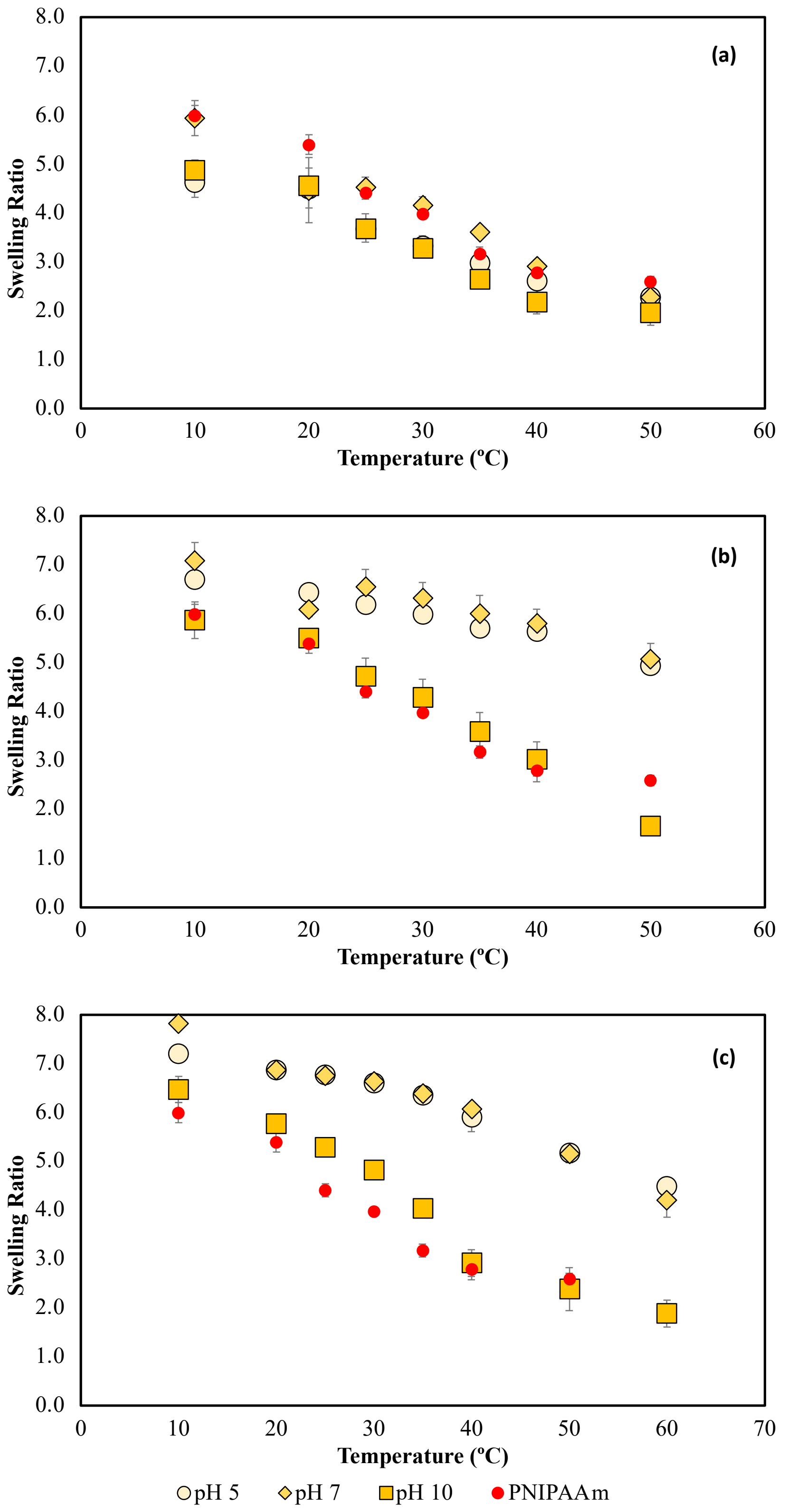

Figure S4. Equilibrium temperature responsive swelling behavior of crosslinked DMAPA hydrogels in various pH buffered aqueous solutions at t = 24 h: **(a)** DMAPA(1) **(b)** DMAPA(5) and **(c)** DMAPA(10). Red circles indicate PNIPAAm swelling averages. N = 3, error bars represent +/- STD.

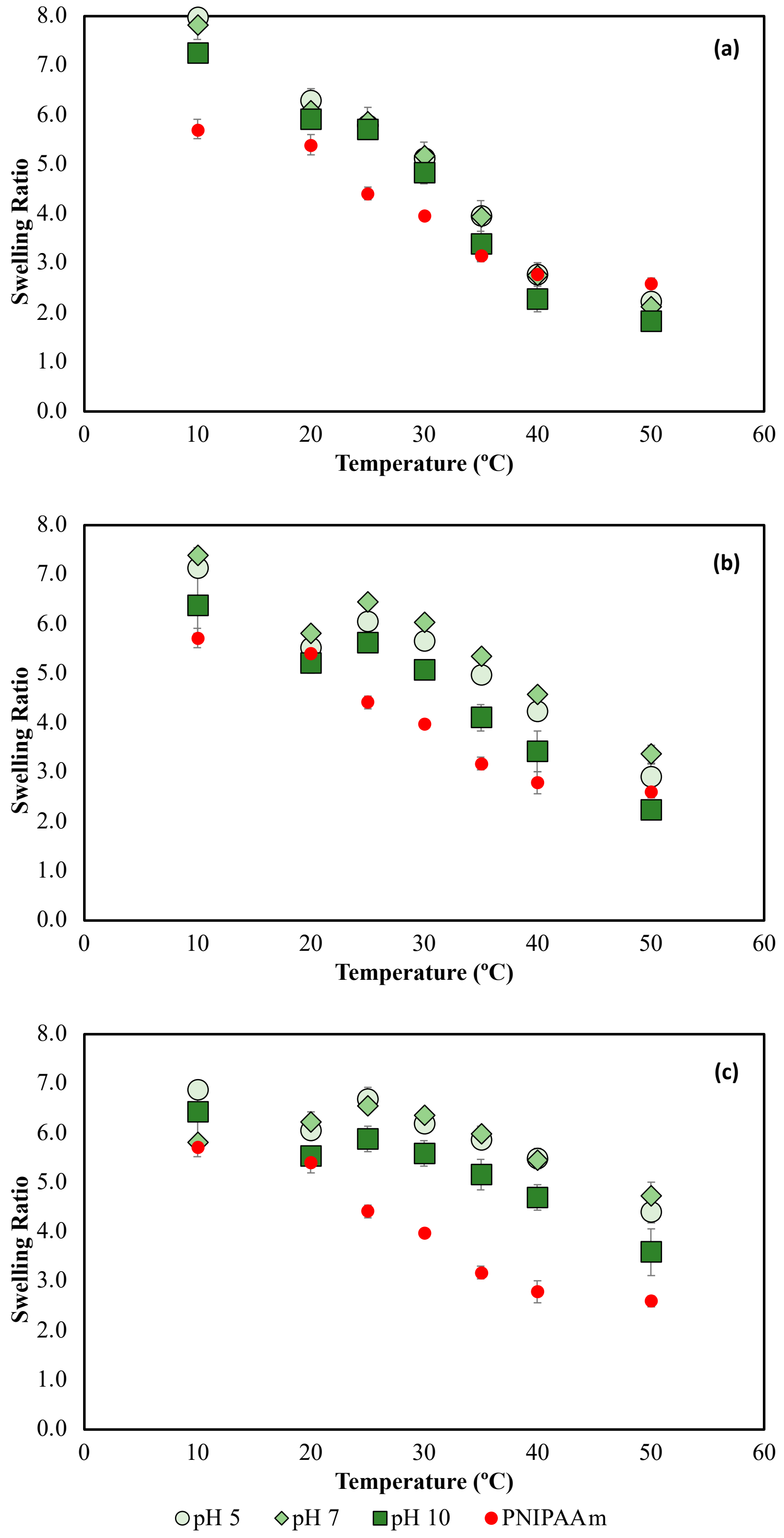

Figure S5. Equilibrium temperature responsive swelling behavior of crosslinked DMAPAQ hydrogels in various pH buffered aqueous solutions at t = 24 h: (a) DMAPAQ(1) (b) DMAPAQ(5) and (c) DMAPAQ(10). Red circles indicate PNIPAAm swelling averages. N = 3, error bars represent +/- STD.
